# Expanded phenotypic diversity of vascular smooth muscle cells in cerebral vessels

**DOI:** 10.1097/JS9.0000000000002387

**Published:** 2025-04-18

**Authors:** Hang Ji, Haogeng Sun, Chao You, Yi Liu

**Affiliations:** Department of Neurosurgery, West China Hospital, Sichuan University, Chengdu, China

Cardiovascular diseases (CVDs) are prevalent worldwide and pose significant threats to human health and well-being. Recent studies highlight the role of vascular smooth muscle cell (vSMC) dedifferentiation in cardiovascular diseases, including atherosclerosis and aortic aneurysms^[^1,2^]^, while the cell complexity in human remain less understood. Single-cell RNA-sequencing (scRNA-seq) has emerged as a primary methodology for elucidating the complexity of cell types and states. Notably, recent studies defined the phenotypes of vSMCs as contractile (*MYH11, TAGLN, ACTA2*), mesenchymal-like (*KLF4, CD34, CD44*), fibroblast-like (*FN1, BGN, DCN*), macrophage-like (*CD68, LGALS3*), osteogenic-like (*SOX9, MSX2*), adipocyte-like (*UCP1, ELOVL3, ADIPOQ*)^[^2,3^]^. These characterizations are primarily based on peripheral arteries and peripheral artery-derived vSMC cell lines. A recent study of murine SMCs further exhibited disparities in gene expression profiles in proximal aortic (*Dcn, Des, Fbln1*), distal aortic (*Pkp4, Mylk4, Foxc2*), and arterial vSMCs (*Acta2, Olfr78*)^[^4^]^, enlightening that the phenotypes of vSMCs may be more sophisticated than previously understood. In addition, some forms of CVDs have a marked predilection for specific sites. For instance, aneurysms and Moyamoya disease occur more frequently in intracranial vessels. These differences prompt us to consider whether, in addition to variations in hemodynamics and possible extravascular mechanical support, there might also be intrinsic regional heterogeneity within the arteries.

Our previous unpublished study integrated scRNA-seq data of 45 vessel samples comprising over 260,000 cells, exhibiting that vSMCs from the normal cerebrovasculature exhibit distinct characteristics compared to those from peripheral. Nevertheless, this work has faced considerable scrutiny. One key point of contention is the utility of the Harmony algorithm for batch effect correction, as it may not efficiently correct sample batch effects on the scale of hundreds of thousands of cells^[^5^]^.

**HIGHLIGHTS**

- A pan-artery single-cell atlas encompassing 227,450 cells from six regions was constructed.
- The vSMCs from cerebral vessels are characterized by *MUSTN1, TNC* and *MINOS1*, and exhibit distinct expression profiles in comparison to those from peripheral arteries.

Herein, we performed scVI, a scalable and flexible generative model designed for large datasets^[^6^]^, for carefully chosen normal cerebral and peripheral vascular scRNA-seq datasets encompassing 227,450 quality-controlled cells from 31 normal human vessel samples (Fig. 1A, Supplemental Table 1, http://links.lww.com/JS9/E65). The sample type level and sample level UMAP embeddings show a satisfactory performance of batch correction (Fig. 1B). Notably, the distribution of cerebral vSMCs was different from that of the peripheral vSMCs, including those from the brachial artery that is also muscular artery (Fig. 1C). Such distinction in clustering was not observed in vascular endothelial cells, indicating a variation of expression profiles between cerebral and peripheral vSMCs. On average, the peripheral vSMCs express a comparable level of *MYH11*, whereas *MUSTN1* was expressed at a higher level in cerebral vSMCs (Fig. 1D). For validation, we integrated additional 13 mouse cerebral and peripheral vascular samples encompassing 48,249 quality-controlled cells based on the Harmony algorithm. We found that cerebral vessel cells remained different from peripheral in the UMAP embedding at the sample type and sample levels (Fig. 1E). Despite that peripheral vSMCs formed one major mixed cluster, the cerebral vSMCs were clustered into separated islands (Fig. 1F). Furthermore, we calculated the differentially expressed genes (DEGs) for each subtype of vSMCs using the MAST algorithm. As a result, cerebral vSMCs robustly expressed genes such as *TNC, MINOS1, PRKCDBP*, and *PLCG2*, while the peripheral vSMCs generally exhibited more intersected DEGs (Fig. 1G). Functional enrichment analysis based on biological process of Gene Ontology revealed that muscle cell-related terms were enriched in brachial vSMCs, while terms enriched in cerebral vSMCs displayed diversity and were associated neuroactivity (Fig. 1H, Supplemental Table 2, http://links.lww.com/JS9/E65), possibly because that the cerebral vascular samples included arteries, veins, and capillaries^[^7^]^.

**Figure 1.**
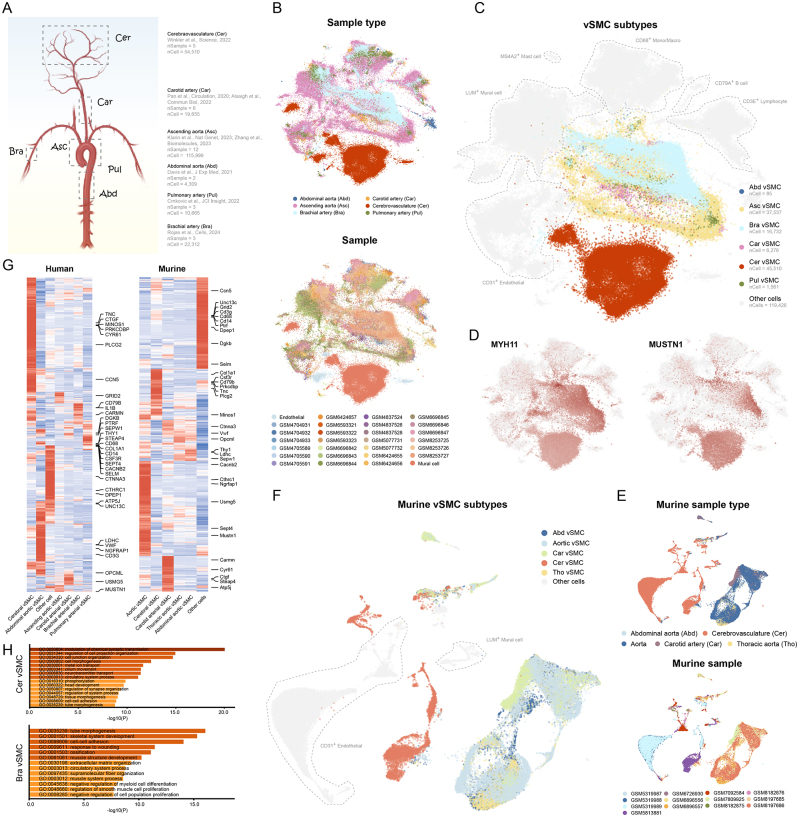
Cerebral vSMCs represents distinct phenotype of vSMCs. (**A**) Samples included in this study with corresponding sources and cell counts. The diagram was generated using the Biorender webtool (https://www.Biorender.Com/learn). (**B**) Sample type level (upper panel) and sample level (lower panel) UMAP embeddings of the human vascular scRNA-seq datasets. Batch effects were corrected using the scVI. GEO accessions of each sample were shown. The scRNA-seq datasets of normal cerebrovasculature from Winkler et al. Were retrieved from https://adult-brain-vasc.Cells.Ucsc.Edu, and were shown as Endothelial (nSample = 5) and Mural cell (nSample = 5) for these datasets were given as processed, integrated expression matrices. (**C**) UMAP embedding of vSMCs (*MYH11, TAGLN, ACTA2*) from different sample types. (**D**) The expression of classical vSMC marker gene *MYH11* and *MUSTN1* that enriched in cerebral vSMCs in different sources of vSMCs. (**E**) Sample type level and sample level UMAP embeddings of the mouse vascular scRNA-seq datasets. Batch effects were corrected using Harmony. A total of 13 samples from 8 studies were included (GSE174564, GSE193533, GSE220514, GSE221789, GSE224246, GSE227157, GSE262996, GSE263739). A total of 48 249 cells passed quality-control and doublet filtering procedures. (**F**) UMAP embedding of mouse vSMCs from different sample types. (**G**) Differentially expressed genes were calculated using the MAST algorithm and exhibited. (**H**) Functional enrichment analysis based on the biological process terms of Gene Ontology using the webtool Metascape.

Collectively, our results suggest distinct gene expression patterns between cerebral and peripheral vSMCs, which may involve multiple mechanisms. For instance, cerebral vessels must maintain the autoregulation of cerebral blood flow and the high expression of *MUSTN1* enhances cytoskeletal stability and ensures the capacity for sustained tension. Additionally, *CTGF*/*CYR61* integrates TGF-β and Wnt signaling pathways to promote the expression of tight junction proteins such as claudin-5, thereby supporting the function of brain-blood barrier. Meanwhile, peripheral vSMCs exhibit high levels of contraction phenotype markers (e.g., *MYH11*), which enable rapid responses to blood pressure fluctuations. Peripheral vSMCs are also prone to phenotypic transformation induced by PDGF-BB, promoting neointima formation. Together, these results indicate an expanded phenotypic spectrum of vSMCs by previously recognized six shades.

There are also limitations, particularly our analysis merely relies on transcriptomic data. Therefore, further functional validations are essential for confirming these findings.

## Data and code availability statement

The raw data and R codes can be currently accessed from the corresponding author upon reasonable request.

## References

[1] Pan H Ho SE Xue C. Atherosclerosis is a smooth muscle cell-driven tumor-like disease. Circulation 2024;149:1885–98. 38686559 10.1161/CIRCULATIONAHA.123.067587 PMC11164647

[2] Yap C Mieremet A de Vries CJM Micha D de Waard V. Six shades of vascular smooth muscle cells illuminated by KLF4 (Krüppel-Like Factor 4). Arterioscler Thromb Vasc Biol 2021;41:2693–707. 34470477 10.1161/ATVBAHA.121.316600 PMC8545254

[3] Bennett MR Sinha S Owens GK. Vascular smooth muscle cells in atherosclerosis. Circ Res 2016;118:692–702. 26892967 10.1161/CIRCRESAHA.115.306361 PMC4762053

[4] Muhl L Mocci G Pietilä R. A single-cell transcriptomic inventory of murine smooth muscle cells. Dev Cell 2022;57:2426–43.e6. 36283392 10.1016/j.devcel.2022.09.015

[5] Luecken MD Buttner M Chaichoompu K. Benchmarking atlas-level data integration in single-cell genomics. Nat Methods 2022;19:41–50. 34949812 10.1038/s41592-021-01336-8 PMC8748196

[6] Lopez R Regier J Cole MB Jordan MI Yosef N. Deep generative modeling for single-cell transcriptomics. Nature Methods 2018;15:1053–58. 30504886 10.1038/s41592-018-0229-2 PMC6289068

[7] Winkler EA Kim CN Ross JM. A single-cell atlas of the normal and malformed human brain vasculature. Science 2022;375:eabi7377. 35084939 10.1126/science.abi7377 PMC8995178

